# Interpreting findings from Mendelian randomization using the MR-Egger method

**DOI:** 10.1007/s10654-017-0255-x

**Published:** 2017-05-19

**Authors:** Stephen Burgess, Simon G. Thompson

**Affiliations:** 1 MRC Biostatistics Unit, Cambridge Institute of Public Health, University of Cambridge, Forvie Site, Robinson Way, Cambridge, CB2 0SR UK; 2 Department of Public Health and Primary Care, University of Cambridge, Cambridge, UK

**Keywords:** Mendelian randomization, Instrumental variable, Robust methods, MR-Egger, Summarized data

## Abstract

Mendelian randomization-Egger (MR-Egger) is an analysis method for Mendelian randomization using summarized genetic data. MR-Egger consists of three parts: (1) a test for directional pleiotropy, (2) a test for a causal effect, and (3) an estimate of the causal effect. While conventional analysis methods for Mendelian randomization assume that all genetic variants satisfy the instrumental variable assumptions, the MR-Egger method is able to assess whether genetic variants have pleiotropic effects on the outcome that differ on average from zero (directional pleiotropy), as well as to provide a consistent estimate of the causal effect, under a weaker assumption—the InSIDE (INstrument Strength Independent of Direct Effect) assumption. In this paper, we provide a critical assessment of the MR-Egger method with regard to its implementation and interpretation. While the MR-Egger method is a worthwhile sensitivity analysis for detecting violations of the instrumental variable assumptions, there are several reasons why causal estimates from the MR-Egger method may be biased and have inflated Type 1 error rates in practice, including violations of the InSIDE assumption and the influence of outlying variants. The issues raised in this paper have potentially serious consequences for causal inferences from the MR-Egger approach. We give examples of scenarios in which the estimates from conventional Mendelian randomization methods and MR-Egger differ, and discuss how to interpret findings in such cases.

**Electronic supplementary material:**

The online version of this article (doi:10.1007/s10654-017-0255-x) contains supplementary material, which is available to authorized users.

## Introduction

The technique of Mendelian randomization has become a widely-used part of the epidemiologist’s toolkit for investigating causal relationships between risk factors and outcomes using observational data [1, 2]. Although in some cases, the necessary assumption that genetic variants are valid instrumental variables is credible, in other cases the instrumental variable assumptions are not plausible [3]. The instrumental variable assumptions state that a genetic variant used in a Mendelian randomization investigated must be:

1. associated with the risk factor;
2. not associated with any confounder of the risk factor–outcome association;
3. not associated with the outcome conditional on the risk factor and confounders [4, 5].

This implies that there is no alternative causal pathway from the genetic variant to the outcome except for that via the risk factor [6]. These assumptions may be reasonable when the risk factor is a protein biomarker and the genetic variants are located in or around the coding region for that protein. In such a case, causal inferences from a straightforward application of instrumental variable methods have some credibility. However, they are more questionable for polygenic risk factors, such as body mass index or blood pressure, as the influence of genetic variants on such a risk factor is unlikely to be specific [7, 8].

Mendelian randomization-Egger (MR-Egger) is a statistical method that can be employed when the instrumental variable assumptions do not hold, but a weaker assumption is satisfied [9]. The method is being increasingly used in practice, with applications including analyses of the causal effects of plasma urate on coronary heart disease risk [10], of height on income [11], of sleep patterns on type 2 diabetes [12], and of pubertal development on prostate cancer risk [13]. However, critical assessment of the method is lacking. In this paper, we first discuss implementation of the MR-Egger method. We then provide guidance to the applied practitioner in what circumstances the method will give reasonable estimates, and how to interpret these estimates, particularly for cases where the MR-Egger and conventional methods for Mendelian randomization give different results.

## Implementation of the MR-Egger method

MR-Egger consists of three parts: (1) a test that indicates both violations of the instrumental variable assumptions and bias in conventional instrumental variable analysis methods; (2) a test for a causal effect; and (3) an estimate of the causal effect. Software code in R for implementing all of the analyses in this paper is provided in “Appendix A.1” in supplementary material.

### Assumed framework of data and genetic associations

We assume that all relationships between variables (in particular, the genetic associations with the risk factor and with the outcome, and the causal effect of the risk factor on the outcome) are linear with no effect modification. We also assume that all genetic variants are uncorrelated (that is, not in linkage disequilibrium), although conventional instrumental variable methods for analysing summarized data from correlated variants have been developed [14], and similar extensions to the MR-Egger method are discussed later in this paper. The association between genetic variant $G_j$ ($j = 1, 2, \ldots , J$) and the outcome is denoted $\beta _{Yj}$, and the association between genetic variant $G_j$ and the risk factor is denoted $\beta _{Xj}$.

**Fig. 1:**
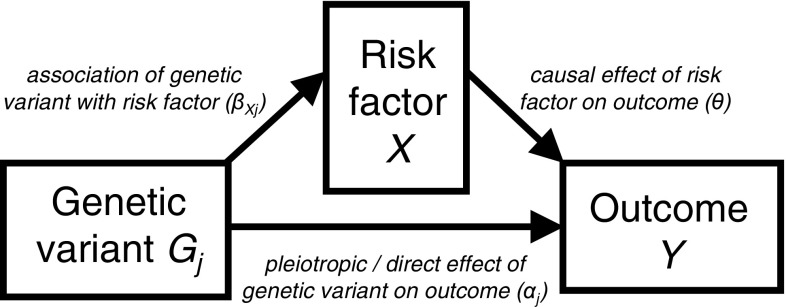
Decomposing association for genetic variant $G_j$ with the outcome into a indirect (causal) effect via the risk factor and an direct (pleiotropic) effect (see Eq. 1)

The genetic association with the outcome can be decomposed into the sum of a direct (pleiotropic) effect and an indirect (causal) effect:

1 $$\begin{aligned} \beta _{Yj} = \alpha _j + \theta \beta _{Xj} \end{aligned}$$

where $\alpha _j$ is the effect of the genetic variant on the outcome that is not mediated via the risk factor of interest, and $\theta$ is the causal effect of the risk factor on the outcome [15]; see Fig. 1. A genetic variant is referred to as pleiotropic if it has associations with more than one risk factor on different causal pathways [16]. Any such effect is included in the parameter $\alpha _j$; a genetic variant is pleiotropic if $\alpha _j \ne 0$. A pleiotropic genetic variant is not a valid instrumental variable.

### Inverse-variance weighted method

With a single genetic variant $G_j$ that satisfies the instrumental variable assumptions, the causal effect of the risk factor on the outcome can be consistently estimated as a simple ratio of association estimates: ${\hat{\theta }_{j}} = \frac{\hat{\beta }_{Yj}}{\hat{\beta }_{Xj}}$ [16], where $\hat{\beta }_{Yj}$ is the estimated coefficient from univariable regression of the outcome on the *j*th genetic variant, and likewise ${\hat{\beta }_{Xj}}$ from univariable regression of the risk factor on the *j*th genetic variant. With multiple genetic variants, the ratio estimates from each genetic variant can be averaged using an inverse-variance weighted formula taken from the meta-analysis literature to provide an overall causal estimate known as the inverse-variance weighted (IVW) estimate [17]. This assumes that the ratio estimates all provide independent evidence on the causal effect; this occurs when the genetic variants are uncorrelated. If the variance terms are taken as $\frac{{{\mathrm{se}}}({\hat{\beta }_{Yj}})^2}{\hat{\beta }_{Xj}^2}$ (this is the first term from a delta method expansion for the ratio estimate [18]), then the pooled estimate (assuming a fixed-effect model) is [19]:

2 $$\begin{aligned} \hat{\theta }_{IVW} = \frac{\sum _j \hat{\beta }_{Yj} \hat{\beta }_{Xj} {{\mathrm{se}}}({\hat{\beta }}_{Yj})^{-2}}{\sum _j \hat{\beta }_{Xj}^2 {{\mathrm{se}}}(\hat{\beta }_{Yj})^{-2}}. \end{aligned}$$

This same estimate is obtained from the two-stage least squares analysis method for individual-level data when the genetic variants are uncorrelated [20]. The same estimate can also be obtained from a weighted linear regression of the genetic associations with the outcome (${\hat{\beta }}_{Yj}$) on the genetic associations with the risk factor (${\hat{\beta }}_{Xj}$) using inverse-variance weights (${{\mathrm{se}}}(\hat{\beta }_{Yj})^{-2}$) when there is no intercept term in the regression model [14]:

3 $$\begin{aligned} \hat{\beta }_{Yj} = \theta _{IVW} \hat{\beta }_{Xj} + \epsilon _{Ij}; \quad \epsilon _{Ij} \sim \mathcal {N}(0, \sigma ^2 {{\mathrm{se}}}(\hat{\beta }_{Yj})^{2}) \end{aligned}$$

where $\hat{\beta }_{Yj}$ and $\hat{\beta }_{Xj}$ are the data in the model, $\theta _{IVW}$ is the parameter, and $\epsilon _{Ij}$ is the residual term. To obtain the same standard error for the causal estimate from the regression analysis as from the fixed-effect meta-analysis, the residual standard error in the regression ($\sigma$) must be set to equal one [21].

If the pleiotropic effects of the genetic variants are all zero ($\alpha _j = 0$ for all *j*; in other words, if all genetic variants are valid instrumental variables), then each of the $\hat{\theta }_j$ will be a consistent estimate of the causal effect, and the overall estimate $\hat{\theta }_{IVW}$ (a weighted mean of the $\hat{\theta }_j$) will be a consistent estimate of the causal effect.

### MR-Egger method

MR-Egger is performed by a simple modification to the weighted linear regression described above. Rather than setting the intercept term to be zero, the term is estimated as part of the analysis [9]:

4 $$\begin{aligned} \hat{\beta }_{Yj} = \theta _{0E} + \theta _{1E} \hat{\beta }_{Xj} + \epsilon _{Ej}; \quad \epsilon _{Ej} \sim \mathcal {N}(0, \sigma '^2 {{\mathrm{se}}}(\hat{\beta }_{Yj})^{2}) \end{aligned}$$

where the parameter $\theta _{0E}$ is the intercept, $\theta _{1E}$ is the slope (MR-Egger estimate), and $\epsilon _{Ej}$ is the residual term. If the intercept term is exactly equal to zero, then the MR-Egger estimate will equal the IVW estimate. Alternatively, if the pleiotropic effects $\alpha _j$ are independently distributed from the genetic associations with the risk factor $\beta _{Xj}$ [22] – this is referred to as the InSIDE assumption (INstrument Strength Independent of Direct Effect) – then the MR-Egger estimate will be a consistent estimate of the causal effect as the sample size and number of genetic variants both increase [9]. For a fixed number of genetic variants, the MR-Egger estimate is a consistent estimate as the sample size increases if the weighted covariance between the $\alpha _j$ and the $\beta _{Xj}$ parameters using the inverse-variance weights ${{\mathrm{se}}}(\hat{\beta }_{Yj})^{-2}$ is exactly zero (see "Appendix A.2" in supplementary material). The test of whether the MR-Egger estimate differs from zero is referred to as the MR-Egger causal test.

Under the InSIDE assumption, the intercept from the MR-Egger analysis can be interpreted as the average pleiotropic effect of a genetic variant included in the analysis (the weighted mean of the $\alpha _j$ using the inverse-variance weights ${{\mathrm{se}}}(\hat{\beta }_{Yj})^{-2}$). If the average pleiotropic effect is zero (known as balanced pleiotropy), then the IVW method gives a consistent estimate of the causal effect (under the InSIDE assumption). Conversely, if the intercept from the MR-Egger analysis is not equal to zero, then either the average pleiotropic effect differs from zero (known as directional pleiotropy) or the InSIDE assumption is violated (or both). Hence, testing the intercept from the MR-Egger analysis provides an assessment of the validity of the instrumental variable assumptions, with a non-zero intercept indicating that the IVW estimate is biased. The test of whether the intercept differs from zero is referred to as the MR-Egger intercept test.

### Intuitive motivation for MR-Egger analysis

**Fig. 2:**
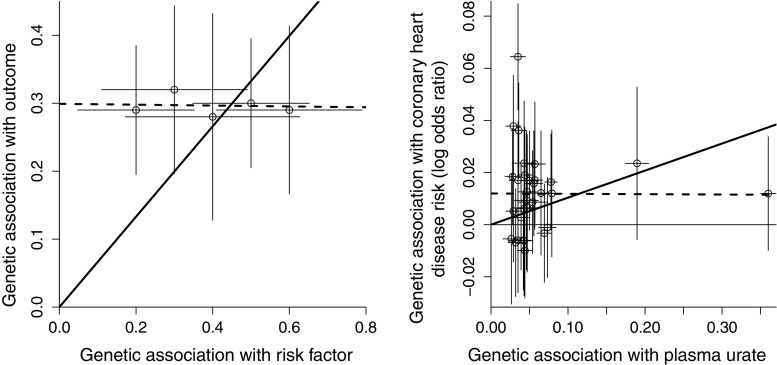
Graph showing simulated (*left panel*) and real-data (*right panel*) examples in which inverse-variance weighted estimate (*solid line*) and MR-Egger estimate (*dashed line*) differ substantially. *Each point* represents the per allele associations of a single genetic variant (*lines* from *each point* are 95% confidence intervals for the associations). In both cases, the inverse-variance weighted estimate is positive, whereas the MR-Egger causal estimate is null with intercept term differing from zero

Figure 2 provides two examples where the estimates from the IVW and MR-Egger methods differ substantially. The left panel of Fig. 2 is a simulated illustration, whereas the right panel is a real-data example where the risk factor is plasma urate and the outcome is coronary heart disease (CHD) risk [10] (the choice of genetic variants and the associations with plasma urate are taken from White et al. [10]; associations with CHD risk are taken from the CARDIoGRAMplusC4D consortium 2015 data release [23], see Web Table A1 in supplementary material). The horizontal axis of the graph displays the estimated genetic associations with the risk factor ($\hat{\beta }_{Xj}$); the vertical axis displays estimated genetic associations with the outcome ($\hat{\beta }_{Yj}$). Each point on the graph represents a single genetic variant; lines represent 95% confidence intervals for the genetic associations. For any individual genetic variant, the ratio estimate $\hat{\theta }_j$ is the gradient of the line connecting the relevant datapoint to the origin. The IVW estimate (solid line) is a weighted mean of these ratio estimates.

Although all five of the genetic variants in Fig. 2 (left panel) individually suggest a positive causal effect of the risk factor on the outcome, a dose–response relationship in the associations is absent. Genetic variants that have a greater magnitude of association with the risk factor do not also have a greater magnitude of association with the outcome. This is contrary to what would be expected if the associations of the genetic variants with the outcome were entirely mediated via the risk factor, and hence it is unlikely that all of the genetic variants are valid instrumental variables. While the individual ratio estimates are all positive (as is the IVW estimate), the regression model from MR-Egger (dashed line) tells a different story. The intercept term from MR-Egger regression differs from zero, and the causal estimate from MR-Egger is compatible with the null. This suggests that the set of genetic variants suffers from directional pleiotropy and, once this pleiotropy is accounted for, there is no residual evidence for a causal effect. A similar situation applies to the example of plasma urate and CHD risk in Fig. 2 (right panel) [24]. In contrast, the simple and weighted median methods of Bowden et al. [25] would give similar estimates to the IVW method in Fig. 2 (left panel). In Fig. 2 (right panel), estimates from the different methods (odds ratio per 1 standard deviation increase in plasma urate with 95% confidence interval) are 1.11 (1.03, 1.20) for the IVW method, 1.00 (0.90, 1.10) for the MR-Egger method, 1.05 (0.99, 1.11) for the weighted median method, and 1.20 (1.07, 1.34) for the simple median method.

A conventional Mendelian randomization analysis—defined as an analysis in which the instrumental variable assumptions are assumed to hold for all of the genetic variants—assesses whether genetic variants that are associated with the risk factor also associate with the outcome. Median-based methods assess whether the majority (or weighted majority) of genetic variants are associated with the outcome. In comparison, MR-Egger assesses whether there is a dose–response relationship between the genetic associations with the risk factor and those with the outcome.

### Orientation of the genetic variants

Genetic associations are usually the per allele associations of the genetic variants with the risk factor and with the outcome. Associations of genetic variants (assumed here to be single nucleotide polymorphisms, SNPs, although other polymorphisms could also be considered) can be quoted with respect to either the major or the minor allele. For example, if a genetic variant has a C allele and a T allele, the association could equivalently be given as (say) 0.243 units per additional copy of the C allele, or as $-0.243$ units per additional copy of the T allele. There is no prior reason why one orientation should be preferred over the other.

**Fig. 3:**
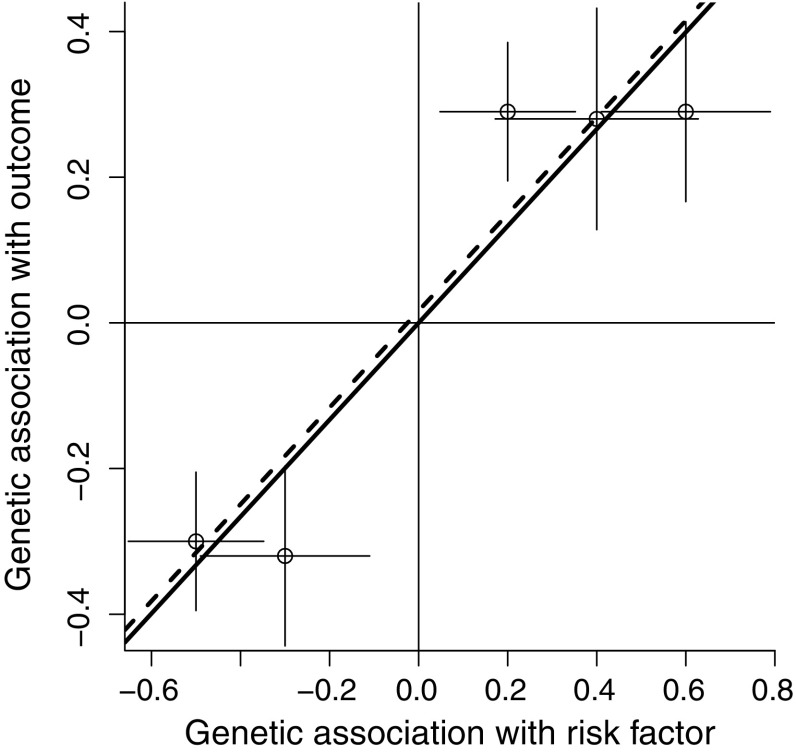
Graph showing same simulated example as in Fig. 2 (*left panel*), except that three variants are positively orientated and two negatively. The inverse-variance weighted estimate (*solid line*) is unaffected by the orientation of variants, whereas the MR-Egger estimate (*dashed line*) is affected by the choice of orientation, with the intercept term attenuating and the MR-Egger estimate approaching the inverse-variance weighted estimate

Figure 3 displays associations for the same variants as in Fig. 2 (left panel), except that three of the variants are positively orientated (orientated with respect to the risk factor-increasing allele), and two are negatively orientated. For the IVW estimate (solid line), the lack of intercept in the regression model means that a genetic variant provides exactly the same contribution to the analysis when orientated with respect to the either allele. However, in the MR-Egger analysis (dashed line), changing the orientation of any one genetic variant will change the definition of the pleiotropic effect $\alpha _j$ (Eq. 1), and also the assessment of directional pleiotropy and the InSIDE assumption itself.

To address this issue, we orientate the genetic variants so that the associations with the risk factor all have the same sign. This means that directional pleiotropy is defined with respect to the risk factor-increasing allele (or equivalently, the risk factor-decreasing allele). Orientating the genetic variants in this way means that the MR-Egger analysis does not depend on the original coding of the genetic variants, and directional pleiotropy is perhaps more likely to be detected. It will be detected if pleiotropic effects tend to act in a consistent direction that corresponds to increases (or decreases) in the risk factor (particularly if the InSIDE assumption is additionally violated and genetic variants having greater associations with the risk factor also have larger pleiotropic effects).

As genetic variants included in a Mendelian randomization analysis are usually chosen as those having statistically robust associations with the risk factor, it is unlikely that the identity of the risk factor-increasing allele for a genetic variant is uncertain. However, if a genetic variant has a weak association with the risk factor, then a small change in its association with the risk factor from positive to negative will change its orientation in the MR-Egger analysis, thus potentially having a large impact on the MR-Egger causal estimate and intercept terms. This situation may arise if the genetic variants are chosen with respect to one variable, but associations are estimated with respect to a related variable (for example, genetic variants are chosen on the basis of their association with body mass index, but the risk factor of interest is a site-specific measure of adiposity) or are estimated in another population (for example, genetic variants are chosen on the basis of their association in European-descent individuals, but the associations used in the analysis are estimated in African-descent individuals). It may be prudent in such a situation to orientate variants according to their associations in the larger dataset.

## Interpretation of results from the MR-Egger method

In this section, we present issues relating to the interpretation of results from the MR-Egger method, including the precision of estimates, influence of outlying variants, violations of the InSIDE assumption, and situations where the MR-Egger and conventional methods give differing results.

### Precision of the MR-Egger estimate

In the IVW method, the estimated variance of the causal estimate from the regression analysis is proportional to the weighted sum of the squares of the $\hat{\beta }_{Xj}$ estimates:

5 $$\begin{aligned} \text{ Variance } \text{ of } \text{ the } \text{ IVW } \text{ estimate } = \frac{\hat{\sigma }^2}{\sum _j \hat{\beta }_{Xj}^2 {{\mathrm{se}}}(\hat{\beta }_{Yj})^{-2}} \end{aligned}$$

where $\hat{\sigma }$ is the estimated residual standard error from Eq. (3); this is 1 when using a fixed-effect meta-analysis model for combining the ratio estimates, corresponding to the assumption of no heterogeneity in the causal estimates from the individual genetic variants [26]. A random-effects model that allows for multiplicative overdispersion in these causal estimates should be preferred if there is suspicion of potential pleiotropy. This can be achieved by estimating the residual standard error as part of the analysis (a multiplicative random-effects model); an estimate of the residual standard error above 1 corresponds to overdispersion of the genetic associations [21]. However, if the estimate of the residual standard error is less than 1, then this is not plausible (for uncorrelated variants), as there is no biological mechanism that would lead to underdispersion of the genetic associations. Therefore standard errors of the regression coefficients should be corrected by dividing by the minimum of the residual standard error estimate and one, so that a random-effects model can never give more precise estimates than a fixed-effect model. Underdispersion may be a sign that the genetic variants may have been chosen inappropriately in a way that preferentially selects similar variants. However, it may simply be a chance finding.

In the MR-Egger method, the variance of the causal estimate is inversely proportional to the weighted variance of the $\hat{\beta }_{Xj}$ estimates:

6 $$\begin{aligned} &\text{ Variance } \text{ of } \text{ the } \text{ MR-Egger } \text{ estimate } \\&\quad = \frac{\hat{\sigma }'^2}{\sum _j (\hat{\beta }_{Xj} - \bar{\beta }_{X})^2 {{\mathrm{se}}}(\hat{\beta }_{Yj})^{-2}}. \end{aligned}$$

where $\hat{\sigma }'$ is the estimated residual standard error from Eq. (4), and $\bar{\beta }_{X}$ is the weighted average association with the risk factor amongst the genetic variants (using the inverse-variance weights ${{\mathrm{se}}}(\hat{\beta }_{Yj})^{-2}$) [27]. As pleiotropic effects of genetic variants will lead to overdispersion in the MR-Egger regression model, heterogeneity between the causal estimates is expected, and so a random-effects analysis should always be preferred when using MR-Egger. If heterogeneity is absent, then a random-effects analysis is equivalent to a fixed-effect analysis.

**Fig. 4:**
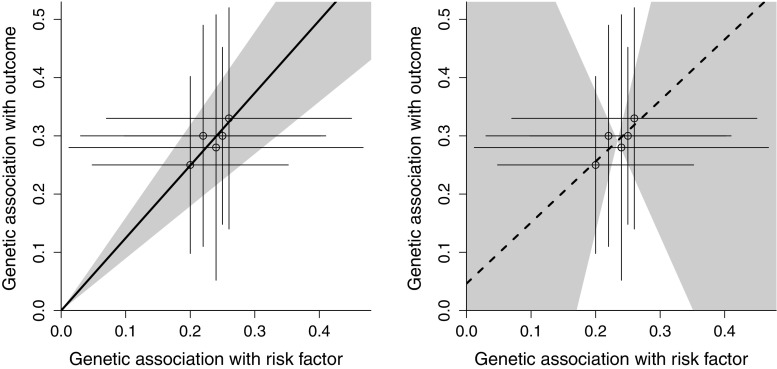
Graph showing hypothetical example in which genetic associations with the risk factor and with the outcome are similar for all variants. *Left panel* inverse-variance weighted estimate (*solid line*) and 95% confidence interval (*grey area*) suggest strong evidence for a positive causal effect. *Right panel* MR-Egger estimate (*dashed line*) and 95% confidence interval (*grey area*) suggest no evidence against the instrumental variable assumptions (intercept test), but also no evidence for a causal effect (causal test)

While the precision of the IVW estimate depends on the proportion of variance in the risk factor explained by the genetic variants (typically measured by the $R^2$ statistic) [28], the precision of the MR-Egger estimate additionally depends on the variability between the genetic associations with the risk factor [29]. In a hypothetical case where several genetic variants have almost equal associations with the risk factor, the IVW estimate may be very precise, particularly if the associations with the outcome are similar to each other (Fig. 4; left panel—grey area represents 95% confidence interval for the IVW estimate). However, in the MR-Egger analysis, the precisions of the intercept and causal estimates will be low (Fig. 4; right panel—grey area represents 95% confidence interval for the MR-Egger intercept and causal estimate). This behaviour can be diagnosed using the $I^2$ statistic from the meta-analysis literature as proposed by Bowden et al. [29]. The Bowden $I^2$ statistic is a measure of instrument strength for the MR-Egger method; values close to one indicate that the MR-Egger estimate does not suffer from ‘weak instrument bias’. In fact, if the genetic associations with the risk factor are exactly equal, then neither parameter in the MR-Egger regression model is formally identified, and the $I^2$ statistic is zero.

As an illustration of this, despite broad consistency of the causal estimates across the genetic variants in Fig. 4 (right panel), the MR-Egger analysis is not able to reliably detect a dose–response relationship in the genetic associations with the risk factor and with the outcome, and hence cannot distinguish between pleiotropy and a causal effect.

The standard error of the causal estimate from the MR-Egger method will typically be larger than that from the IVW method; this will always be the case for fixed-effect analyses. A precise MR-Egger estimate requires genetic variants having a wide range of associations with the risk factor. However, as we discuss next, if one genetic variant has a much stronger association with the risk factor than others, then this variant will have a large influence on the coefficients in the MR-Egger regression.

### Influence of outlying variants on MR-Egger estimates

**Fig. 5:**
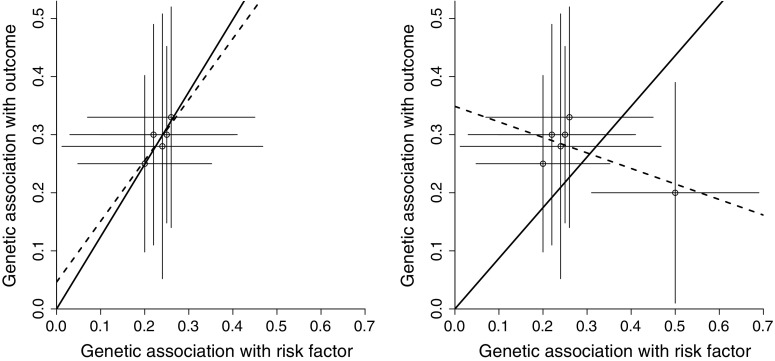
Graph showing same hypothetical example as Fig. 4 (*left panel*) except for the addition of a single extra genetic variant (*right panel*). *Left panel* inverse-variance weighted estimate (*solid line*) and MR-Egger estimate (*dashed line*) are similar. *Right panel* inverse-variance weighted estimate (*solid line*) and MR-Egger estimate (*dashed line*) are markedly different, as the influential genetic variant changes the sign of the MR-Egger estimate

In any regression model, an individual datapoint can have a large influence on the regression coefficients. In Fig. 5 (left and right panels), we see how the addition of a single genetic variant can reverse the sign of the MR-Egger estimate, and lead to rejection of the MR-Egger intercept test. The influence on the IVW estimate is less severe. This scenario is particularly likely for a risk factor such as body mass index, where the lead variant in one gene region (the *FTO* gene region) has a much stronger association with the risk factor than other variants [30]. Influential points can be detected by standard regression diagnostic tools, such as calculating Cook’s distances and/or Studentized residuals for all the datapoints [31], and performing a leave-one-out analysis [32]. Cook’s distance is a measure of leverage, indicating the influence of a datapoint on the regression estimates (larger values indicate greater influence). A Studentized residual is a residual from the regression model divided by an estimate of its standard error, indicating the goodness-of-fit in the regression model for that point (larger values indicate more outlying points). A leave-one-out analysis is conducted by leaving each genetic variant out of the Mendelian randomization analysis in turn, conducting *J* analyses each with $J-1$ datapoints.

We calculated Cook’s distances and Studentized residuals for all the variants included in the MR-Egger analysis of plasma urate and CHD risk presented in Fig. 2 (right panel). The genetic variant with both the largest Cook’s distance and Studentized residual was not one of the two variants having the greatest association with plasma urate, but the variant having the strongest association with CHD risk (rs653178, nearest gene *ATXN2*). However, the omission of this variant did not substantially affect the MR-Egger analysis (neither the rejection of the intercept test, nor the failure to detect a causal effect).

### Plausibility and violations of the InSIDE assumption

While the MR-Egger intercept test does not require the InSIDE assumption to be satisfied to detect violations of the instrumental variable assumptions, the interpretation of the intercept as an average pleiotropic effect, as well as the assessment and estimation of a causal effect using MR-Egger, do rely on the InSIDE assumption. Equally, although the primary assumption for the IVW method is that all variants are valid instruments, it also provides consistent estimates when the average pleiotropic effect is zero and the InSIDE assumption is satisfied. Although the initial presentation of the MR-Egger method [9] gave biased estimates with inflated Type 1 error rates when the InSIDE assumption was not satisfied, the bias and Type 1 error inflation were both less than those for the IVW method. However, subsequent simulations have shown that estimates from the MR-Egger method can be more biased and have greater Type 1 error rates compared with the IVW method in settings when pleiotropic effects of multiple genetic variants act through the same confounder [25]. Hence, the InSIDE assumption is crucial to the interpretation of causal inferences from the MR-Egger method in the case of pleiotropy.

Some general plausibility of the InSIDE assumption can be inferred from the observation that genetic associations with different measured variables tend to be uncorrelated with each other, as demonstrated in empirical studies [33]. If all the genetic variants in a Mendelian randomization analysis have pleiotropic effects, but the pleiotropic effects act via unrelated variables that are not confounders of the risk factor–outcome associations, then the InSIDE assumption seems likely to hold. However, if the pleiotropic effects of several variants all act via the same confounder, the pleiotropic effects and instrument strengths will be strongly correlated, as both depend on the magnitude of the associations of the genetic variants with the confounder. Similarly, if a genetic variant has a pleiotropic effect via a confounder, then this will lead to an association with the risk factor (contributing to the instrument strength) and an association with the outcome (contributing to the pleiotropic effect). Crucially, if the effect of the genetic variant on the confounder increases, then its association with both the risk factor and with the outcome will increase. This means that genetic variants with larger effects on confounders will tend to have both larger instrument strengths and larger pleiotropic effects—leading to violation of the InSIDE assumption. It is difficult to imagine how the InSIDE assumption could be satisfied if several genetic variants have pleiotropic effects acting via confounders.

**Fig. 6:**
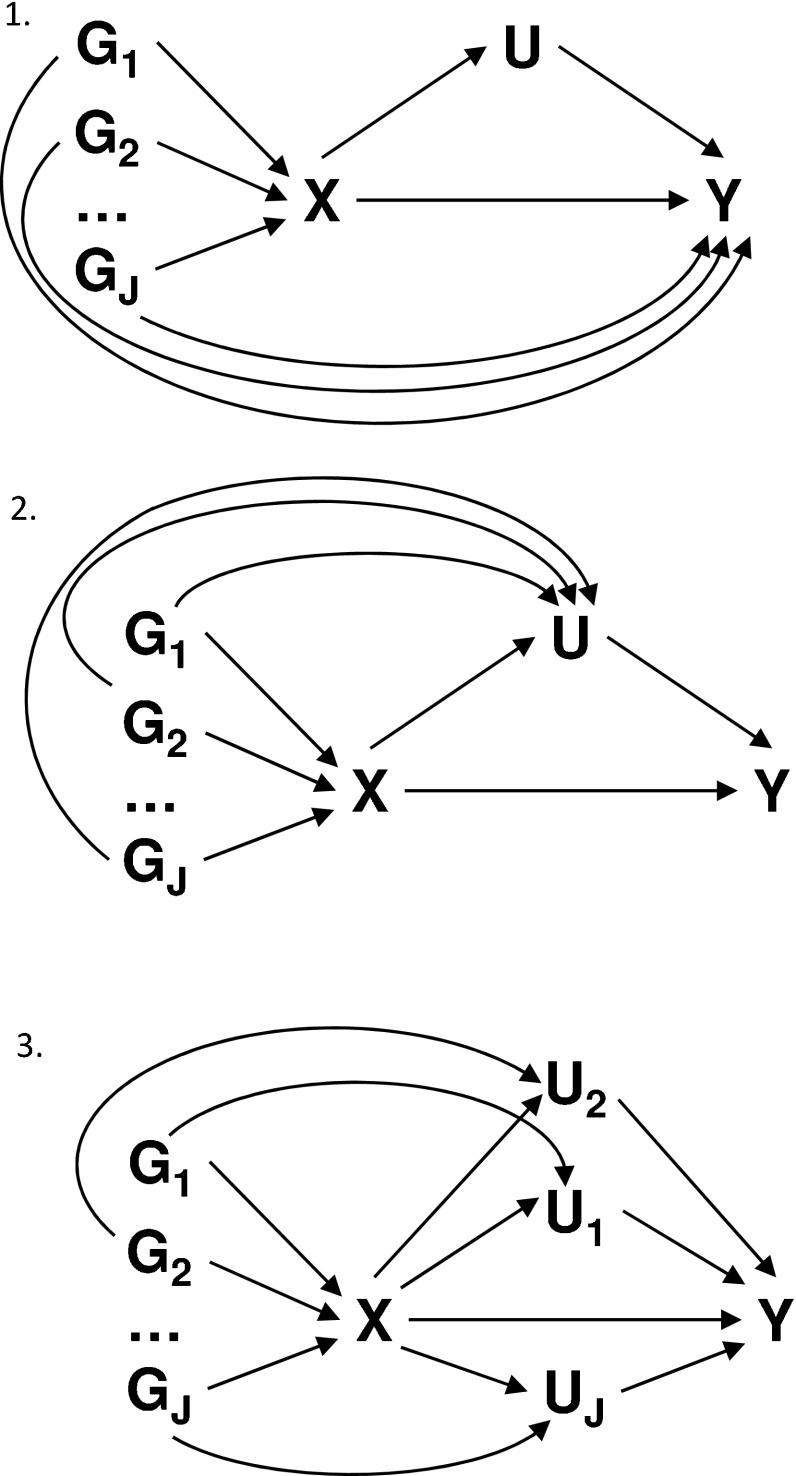
Potential violations of the InSIDE assumption. *Top panel* pleiotropic effects act directly on the outcome (InSIDE satisfied); *middle panel* pleiotropic effects act on the outcome via single confounder (InSIDE violated); *bottom panel* pleiotropic effects act on the outcome via different confounders (InSIDE still violated). *Arrows* from the genetic variants to the risk factor may not be present for all variants; some variants may affect the confounder directly and not the risk factor. *Notation*: $G_1$, $G_2$, $\ldots$, $G_J$, genetic variants; *X*, risk factor; *Y*, outcome; *U*, confounder. Pleiotropic effects are signified by *curved arrows*

Some general plausibility of the InSIDE assumption can be inferred from the observation that genetic associations with different measured variables tend to be uncorrelated with each other, as demonstrated in empirical studies [33]. If all the genetic variants in a Mendelian randomization analysis have pleiotropic effects, but the pleiotropic effects act via unrelated variables that are not confounders of the risk factor–outcome associations, then the InSIDE assumption seems likely to hold (Scenarios 2 and 3 of Bowden et al. [9, 25], Fig. 6 top panel). However, if genetic variants have pleiotropic effects on the outcome that all act via the same confounder, the pleiotropic effects and instrument strengths will be strongly correlated, as both depend on the magnitude of the associations of the genetic variants with the confounder (Scenario 4 of Bowden et al. [9, 25], Fig. 6 middle panel). Further to this, if genetic variants have pleiotropic effects on the outcome that act via different confounders, then the InSIDE assumption will still be violated (Fig. 6 bottom panel). This occurs because, if the effect of the genetic variant on the confounder increases, then its association with both the risk factor (contributing to the instrument strength) and with the outcome (contributing to the pleiotropic effect) will increase. This means that genetic variants with larger effects on confounders will tend to have both larger instrument strengths and larger pleiotropic effects ?—leading to violation of the InSIDE assumption. It is therefore difficult to imagine how the InSIDE assumption could be satisfied if several genetic variants have pleiotropic effects acting via confounders.

It has been claimed that the InSIDE assumption can be empirically tested by assessing the correlation between the ratio estimates for the individual variants and their associations with the risk factor [34]. However, the ratio estimate includes the association with the risk factor as its denominator, so a correlation between the ratio estimates and the associations with the risk factor would be expected even if all genetic variants were valid instruments.

### Comparing results between MR-Egger and conventional Mendelian randomization analyses

**Fig. 7:**
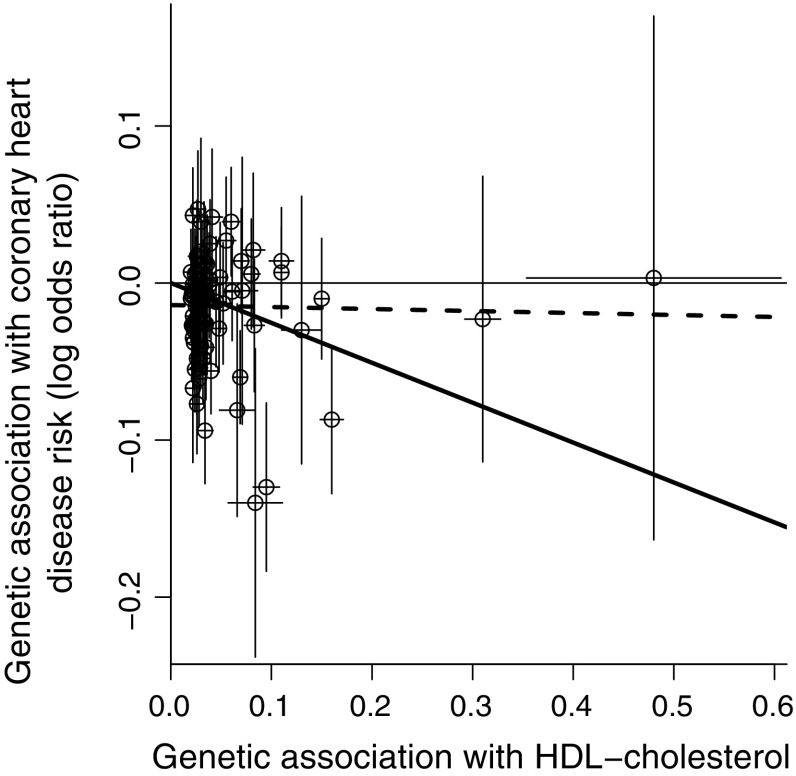
Graph showing further real example in which inverse-variance weighted estimate (*solid line*) and MR-Egger estimate (*dashed line*) differ substantially. *Each point* represents the per allele associations of a single genetic variant (*lines* from *each point* are 95% confidence intervals for the associations). Associations with HDL-cholesterol are in standard deviation units and associations with CHD risk are log odds ratios

An important practical issue for the MR-Egger method is the interpretation of a discordant result from a conventional Mendelian randomization analysis. We have already seen examples in which this can occur: Fig. 2 (left and right panels; positive conventional estimate, null MR-Egger estimate) and Fig. 5 (right panel; positive conventional estimate, negative MR-Egger estimate). Another example is the effect of high-density lipoprotein (HDL) cholesterol on CHD risk using all genome-wide significant variants associated with HDL-cholesterol (including variants known to have pleiotropic effects): the IVW method suggests a protective effect of HDL-cholesterol on CHD risk, whereas the MR-Egger method detects directional pleiotropy and suggests a null causal effect (Fig. 7, details of the analysis are given in the "Appendix A.3" in supplementary material) [25]. Estimates from the different methods (odds ratio per 1 standard deviation increase in HDL-cholesterol with 95% confidence interval) are 0.78 (0.68, 0.89) for the IVW method, 0.99 (0.79, 1.24) for the MR-Egger method, 0.93 (0.92, 1.07) for the weighted median method, and 0.77 (0.64, 0.91) for the simple median method. These examples illustrate the correlation between the intercept term and slope term in MR-Egger regression: if the intercept term is close to zero, then the MR-Egger estimate will be close to the IVW estimate. However, even if the estimates are similar, inferences from the two methods can differ if the MR-Egger estimate is imprecise, as in the example of height on income [11]. In such a case, the MR-Egger analysis does not provide additional evidence for a causal effect, but it does not contradict evidence for a causal effect from a conventional Mendelian randomization analysis either.

Additionally, if the MR-Egger intercept is larger than the association of any of the individual genetic variants (as in Fig. 5, right panel), then this implies (under the InSIDE assumption) that the average pleiotropic effect on the outcome of a genetic variant is larger in magnitude than the observed association with the outcome of all of the individual genetic variants. This seems implausible, and suggests that the InSIDE assumption is likely to be violated. The test for directional pleiotropy indicates that the genetic variants are not all valid instruments, but the negative MR-Egger estimate is highly dubious as the causal estimates from each variant in turn are all positive.

Finally, if a conventional Mendelian randomization analysis suggests no causal effect, then we would be reluctant to consider evidence from the MR-Egger method, as the method was proposed as a sensitivity analysis for a conventional Mendelian randomization analysis. Although it is possible for pleiotropic effects to bias the conventional Mendelian randomization estimate towards the null, it would seem at least as likely for the MR-Egger estimate to be biased due to violations of the InSIDE assumption or due to the influence of strong variants.

## Discussion

In this paper, we have described the problem of pleiotropy in Mendelian randomization, and the potential solution to this problem represented by the MR-Egger method. We have described how to implement the method, its assumptions, and various issues that may bias estimates. Finally, we have discussed how to interpret discordancies between results from the MR-Egger method and those from conventional Mendelian randomization methods.

While the MR-Egger method is a worthwhile sensitivity analysis for Mendelian randomization, it is by no means a panacea for all violations of the instrumental variable assumptions. Several of the issues raised in this paper have potentially serious consequences for MR-Egger estimates. These include violations of the InSIDE assumption—the assumption that the pleiotropic effects of the genetic variants in the analysis are uncorrelated with the associations of the variants with the risk factor. Violations of this assumption have been shown to lead to increased bias and Type 1 error rate inflation in the MR-Egger method compared with conventional methods in realistic simulations [25]. Another serious issue is that of the influence of outlying variants on MR-Egger estimates. We have shown how even a single genetic variant can have a substantial influence on a MR-Egger analysis, leading to rejection of the MR-Egger intercept test and reversal of the sign of the MR-Egger estimate (Fig. 5). A corollary of this is that Mendelian randomization analyses using the MR-Egger method should still seek to use genetic variants that are valid instrumental variables as far as possible.

### Alternative approaches for sensitivity analysis in Mendelian randomization

MR-Egger is far from the only method for sensitivity analysis in Mendelian randomization. Several reviews of such methods exist in the literature [8, 32, 35]. Approaches divide into those for assessing the validity of the instrumental variable assumptions, and robust methods that give consistent estimates of a causal effect under weaker assumptions than those of a conventional Mendelian randomization analysis (such as the MR-Egger method) [32]. Robust methods generally fall into two categories: (1) methods such as MR-Egger, that replace the instrumental variable assumptions with an alternative assumption or assumptions that are assumed to hold for the set of genetic variants (a similar approach for individual-level data was proposed by Kolesár et al. [22]); and (2) overidentification methods that assume the instrumental variable assumptions hold for some of the genetic variants, but not necessarily for all genetic variants. Individual-level data methods based on this approach have been proposed by Kang et al. [36], and Windmeijer et al. [37].

A simple summarized data robust method that falls into the second category is the weighted median method proposed by Bowden et al. [25]. An unweighted median-based analysis proceeds by calculating the causal estimate from each genetic variant individually ($\hat{\theta }_j = \frac{\hat{\beta }_{Yj}}{\hat{\beta }_{Xj}}$), and then calculating the median of these causal estimates. This estimate is consistent for the causal effect provided that at least 50% of the genetic variants are valid instrumental variables, and is unaffected by a few genetic variants with outlying causal estimates. As the sample size increases, the causal estimates from all valid instrumental variables will tend towards the same value, which will equal the median estimate provided that at least 50% of the genetic variants are valid instrumental variables [38]. A weighted median method has also been proposed, in which genetic variants with more precise causal estimates contribute more weight to the analysis [25]. The median-based methods may be more appropriate than the MR-Egger method in scenarios like those in Figs. 4 and 5 if the majority of variants are valid instruments. However, in the scenario in Fig. 2 (left panel), the median-based methods would still suggest a positive causal effect despite evidence for directional pleiotropy.

Another summarized data method that has robustness to outlying variants may be a simple variation of the IVW method using robust regression rather than standard linear regression. For example, regression using MM-estimation with Tukey’s bisquare objective function limits the contribution to the analysis from any single genetic variant [39–41].

No single method should be relied on for causal inference. A causal finding is more reliable if it is corroborated by multiple methods, particularly if the methods make different assumptions [32]. Methods such as MR-Egger are desirable as sensitivity analyses as they allow all genetic variants to violate the instrumental variable assumptions; however they require all genetic variants to satisfy an alternative assumption. In contrast, overidentification methods such as the median-based method allow some genetic variants to violate the instrumental variable assumptions in an arbitrary way, although the majority of variants are assumed to satisfy the assumptions. As such, in applied practice a range of sensitivity analysis should ideally be presented, as well as assessments as to whether the instrumental variable assumptions are satisfied for the genetic variants in the analysis.

### Other violations of the instrumental variable assumptions

Violations of the instrumental variable assumptions in the MR-Egger method are expressed here as pleiotropic effects. However, while all violations of the exclusion restriction assumption (the assumption that the effect of a genetic variant on the outcome only operates via the risk factor; this is equivalent to the third instrumental variable assumption as stated in this paper [6]) can be expressed in terms of pleiotropy [15], other violations cannot be. For example, population stratification is the presence of multiple subpopulations within the sample population [3]. If genetic associations with the risk factor, with the outcome, or the frequency of genetic variants differ between these subpopulations, then there may be a spurious association between the genetic variant and the outcome in the overall population. Such population effects, as well as selection effects (for example, the sample under analysis was ascertained conditional on the risk factor, or else the sample somehow is not representative of the population as a whole), are likely to lead to all genetic variants violating the instrumental variable assumptions, and hence consistency conditions for the robust methods presented above would be unlikely to hold.

### Linearity and homogeneity assumptions

Two assumptions that we have made in the specification of the analysis models for both conventional and MR-Egger methods are those of linearity and homogeneity of the causal effect. These assumptions are not necessary to estimate a causal effect; weaker assumptions (such as monotonicity [42] or a weaker version of the homogeneity assumption [43, 44]) can be made [45]. However, the assumptions of linearity and homogeneity ensure that the same causal effect is identified by all genetic variants that are valid instrumental variables. If the linearity and homogeneity assumptions are violated, then the causal estimate from a single variant still provides a valid test of the causal null hypothesis that the risk factor has no causal effect on the outcome [4]; as does the causal estimate from the IVW method, as this is a linear combination of the causal estimates from the individual variants [14].

We view violations of assumptions that lead to inappropriate inferences (inflated Type 1 error rate of the null hypothesis of no causal effect) as first-order concerns, while violations of assumptions that lead only to inappropriate causal estimates (but appropriate causal inferences both with a null and a non-null causal effect) are viewed as second-order concerns (and questions about the causal estimand, such as those arising due to non-collapsibility with a binary outcome [46], as third-order concerns). Violations of the assumptions of linearity and homogeneity of the causal effect are important, as they affect the interpretation of results from MR-Egger and conventional Mendelian randomization methods, and the applicability of causal estimates in practice. However, they will not lead to inappropriate inferences, and as such are less troublesome than violations of the three core instrumental variable assumptions. There are many reasons why Mendelian randomization estimates may differ from the result of intervening on the risk factor in practice (for example, the mechanism of the intervention, the duration of the intervention, and the timing of the intervening) [47], and so an overly literal interpretation of Mendelian randomization estimates is rarely justified, even when the instrumental variable assumptions are satisfied. An important situation under which the assumptions of linearity and homogeneity are satisfied for the risk factor–outcome relationship is when the causal effect is null.

### Extension to correlated variants

The IVW estimate has previously been extended to account for correlated variants, by fitting the regression model of Eq. (3) using generalized weighted linear regression [14]. Rather than the simple weights ${{\mathrm{se}}}(\hat{\beta }_{Yj})^{-2}$, we use a weighting matrix $\Omega ^{-1}$, where $\Omega$ has elements $\Omega _{j1, j2} = {{\mathrm{se}}}(\hat{\beta }_{Yj1}) {{\mathrm{se}}}(\hat{\beta }_{Yj2}) \rho _{j1, j2}$ and $\rho _{j1, j2}$ is the correlation between the $j_1$th and $j_2$th genetic variants. The IVW estimate accounting for correlation can be calculated either by matrix algebra using the weighting matrix, or by multiplying the genetic associations with the risk factor and outcome by the Cholesky decomposition of the weighting matrix, and then implementing a standard linear regression model with no weighting. A natural extension of the MR-Egger method with correlated variants can be constructed by allowing an intercept term in the generalized weighted linear regression.

With a fixed number of uncorrelated variants, the MR-Egger estimate is consistent when the weighted covariance between the genetic associations with the risk factor and the pleiotropic effects is zero. The analogous result for consistency in the MR-Egger method with correlated variants is provided in "Appendix A.4" in supplementary material. It is unlikely this criterion will be satisfied if all variants are mutually correlated, as correlations between the variants are likely to lead to correlations between the associations with the risk factor and the pleiotropic effects. However, including more than one variant in each gene region can improve precision of the causal estimate [48].

### Conclusion

A typical frustration for statisticians is that their methodological developments are ignored by the applied field. In the case of the MR-Egger method, the opposite situation is true – MR-Egger has been taken up by the field perhaps too rapidly, and often without understanding of the intricacies of the method and its interpretation. While some of the cautions expressed in this paper are also present in the original paper on MR-Egger, others have only come to light following the application of the method, and trying to understand its results. Similar concerns have been raised elsewhere [25, 29, 31, 49].

While we welcome the widespread adoption of MR-Egger, we hope that this paper aids practitioners in its appropriate use and interpretation, and that the method becomes seen rightly as a sensitivity analysis (and a fallible one) for Mendelian randomization, and one of many sensitivity analyses that can (and should) be used to assess the plausibility of any finding from an applied Mendelian randomization investigation.

## Electronic supplementary material

Below is the link to the electronic supplementary material.

Supplementary material 1 (pdf 114 KB)
