# Supplementary material for: Interpreting findings from Mendelian randomization using the MR-Egger method
Source: Eur J Epidemiol. 2017 May 19;32(5):377–89. doi: 10.1007/s10654-017-0255-x (PMC5506233; doi:10.1007/s10654-017-0255-x)
Supplement: Supplementary file 1 — Supplementary material 1 (pdf 114 KB) [file 10654_2017_255_MOESM1_ESM.pdf]

Appendix

A.1 Software code

We provide R code to implement the methods discussed in this paper. The associations of the genetic variants with the risk factor are denoted `betaXG` with standard errors `sebetaXG`. The associations of the genetic variants with the outcome are denoted `betaYG` with standard errors `sebetaYG`. We assume that the genetic variants are independently distributed unless otherwise stated.

Inverse-variance weighted (IVW) method:

```
betaIVW          = sum(betaYG*betaXG*sebetaYG^-2)/sum(betaXG^2*sebetaYG^-2)
sebetaIVW.fixed  = 1/sqrt(sum(betaXG^2*sebetaYG^-2))
```

This is the formula given in equation (2). Equivalently, the inverse-variance weighted estimate can be calculated by weighted linear regression (equation 3):

```
betaIVW                = summary(lm(betaYG~betaXG-1, weights=sebetaYG^-2))$coef[1]
sebetaIVW.fixed        = summary(lm(betaYG~betaXG-1, weights=sebetaYG^-2))$coef[1,2]/
                          summary(lm(betaYG~betaXG-1, weights=sebetaYG^-2))$sigma
sebetaIVW.mult.random = summary(lm(betaYG~betaXG-1, weights=sebetaYG^-2))$coef[1,2]/
                      min(summary(lm(betaYG~betaXG-1, weights=sebetaYG^-2))$sigma,1)
```

In the fixed-effect model, we divide the reported standard error by the estimated residual standard error, to fix the residual standard error to take the value 1 [21]. In the multiplicative random-effects model, we divide by the estimated residual standard error in the case of underdispersion (the variability in the genetic associations is less than would be expected by chance alone). But in the case of overdispersion (that is, heterogeneity of causal effect estimates), no correction is made. The point estimate is unaffected by the choice of a fixed- or multiplicative random-effects model.

As a third alternative, the inverse-variance weighted estimate can be calculated by meta-analysis:

```
library(meta)
betaIVW                = metagen(betaYG/betaXG, abs(sebetaYG/betaXG))$TE.fixed
sebetaIVW.fixed        = metagen(betaYG/betaXG, abs(sebetaYG/betaXG))$seTE.fixed
betaIVW.add.random     = metagen(betaYG/betaXG, abs(sebetaYG/betaXG))$TE.random
sebetaIVW.add.random  = metagen(betaYG/betaXG, abs(sebetaYG/betaXG))$seTE.random
```

While the causal estimates from the fixed-effect and multiplicative random-effects analyses are the same, the estimate from the additive random-effects analysis differs.

Test for directional pleiotropy (intercept test from MR-Egger method):

```
betaYG = betaYG*sign(betaXG); betaXG = abs(betaXG)
interEGGER          = summary(lm(betaYG~betaXG, weights=sebetaYG^-2))$coef[1,1]
seinterEGGER.random = summary(lm(betaYG~betaXG, weights=sebetaYG^-2))$coef[1,2]/
                    min(summary(lm(betaYG~betaXG, weights=sebetaYG^-2))$sigma, 1)
p.dpleio.random     = 2*(1-pt(abs(interEGGER/seinterEGGER.random),df=length(betaXG)-2))
```

The regression model is given in equation (4). Note that the first step in the analysis is to orientate the genetic variants in a consistent way. The `p.dpleio` variable is the p-value for the test of directional pleiotropy. A low p-value indicates rejection of the null hypothesis of balanced (or no) pleiotropy, in favour of the alternative hypothesis that there is directional pleiotropy. The (multiplicative) random-effects analysis is strongly preferred for MR-Egger; random-effects models are also preferred for the inverse-variance weighted method if pleiotropy is suspected.

MR-Egger causal estimate and test of the causal null hypothesis:

```
betaYG = betaYG*sign(betaXG); betaXG = abs(betaXG)
betaEGGER          = summary(lm(betaYG~betaXG, weights=sebetaYG^-2))$coef[2,1]
sebetaEGGER.fixed  = summary(lm(betaYG~betaXG, weights=sebetaYG^-2))$coef[2,2]/
                      summary(lm(betaYG~betaXG, weights=sebetaYG^-2))$sigma
sebetaEGGER.random = summary(lm(betaYG~betaXG, weights=sebetaYG^-2))$coef[2,2]/
                      min(summary(lm(betaYG~betaXG, weights=sebetaYG^-2))$sigma, 1)
sigmaEGGER         = summary(lm(betaYG~betaXG, weights=sebetaYG^-2))$sigma
betaEGGER.lower    = ifelse(sigmaEGGER<1, min(betaEGGER-qnorm(0.975)*sebetaEGGER.random,
                  betaEGGER-qt(0.975,df=length(betaXG)-2)*sebetaEGGER.random*sigmaEGGER),
                  betaEGGER-qt(0.975,df=length(betaXG)-2)*sebetaEGGER.random)
betaEGGER.upper    = ifelse(sigmaEGGER<1, max(betaEGGER+qnorm(0.975)*sebetaEGGER.random,
                  betaEGGER+qt(0.975,df=length(betaXG)-2)*sebetaEGGER.random*sigmaEGGER),
                  betaEGGER+qt(0.975,df=length(betaXG)-2)*sebetaEGGER.random)
p.causal.random    = ifelse(sigmaEGGER<1, max(2*(1-pnorm(abs(betaEGGER/sebetaEGGER.random))),
                 2*(1-pt(abs(betaEGGER/sebetaEGGER.random/sigmaEGGER),df=length(betaXG)-2))),
                 2*(1-pt(abs(betaEGGER/sebetaEGGER.random),df=length(betaXG)-2)))
```

The `p.causal` variable is the p-value for the test of a causal effect. A low p-value indicates rejection of the null hypothesis of no causal effect, in favour of the alternative hypothesis that there is a causal effect of the risk factor on the outcome. The (slightly convoluted) code for constructing a confidence interval and p-value ensures that the confidence interval for the causal estimate is no wider (p-value is no more conservative) using a random-effects analysis compared with a fixed-effect analysis when there is underdispersion (that is, the residual standard error is less than 1). This could occur with small numbers of variants as the random-effects analysis uses a t-distribution for inference, whereas the fixed-effect analysis uses a normal distribution.

Median-based method:

```
weighted.median <- function(betaIV.in, weights.in) {
  betaIV.order  = betaIV.in[order(betaIV.in)]
  weights.order = weights.in[order(betaIV.in)]
  weights.sum   = cumsum(weights.order)-0.5*weights.order
  weights.sum   = weights.sum/sum(weights.order)
         below  = max(which(weights.sum<0.5))
  weighted.est  = betaIV.order[below] + (betaIV.order[below+1]-betaIV.order[below])*
                  (0.5-weights.sum[below])/(weights.sum[below+1]-weights.sum[below])
  return(weighted.est) }

weighted.median.boot = function(betaXG.in, betaYG.in, sebetaXG.in, sebetaYG.in, weights.in){
 # the standard error is estimated based on 1000 bootstrap samples
med = NULL
for(i in 1:1000){
 betaXG.boot = rnorm(length(betaXG.in), mean=betaXG.in, sd=sebetaXG.in)
 betaYG.boot = rnorm(length(betaYG.in), mean=betaYG.in, sd=sebetaYG.in)
 betaIV.boot  = betaYG.boot/betaXG.boot
```

```
 med[i] = weighted.median(betaIV.boot, weights.in)
 }
return(sd(med)) }

betaIV             = betaYG/betaXG
weights            = rep(1, length(betaXG)) # unweighted median
betaSIMPLEMED      = weighted.median(betaIV, weights)
sebetaSIMPLEMED    = weighted.median.boot(betaXG, betaYG, sebetaXG, sebetaYG, weights)

betaIV             = betaYG/betaXG
weights            = (sebetaYG/betaXG)^-2   # weighted median using inverse-variance weights
betaWEIGHTEDMED    = weighted.median(betaIV, weights)
sebetaWEIGHTEDMED = weighted.median.boot(betaXG, betaYG, sebetaXG, sebetaYG, weights)
```

This is an alternative robust analysis method to MR-Egger based on summarized data that gives consistent estimates under the condition that at least 50% of genetic variants are valid instrumental variables (for the weighted median, that at least 50% of the weight is from valid instrumental variables). See Bowden et al. [25] for more details.

Robust IVW method:

The inverse-variance weighted (IVW) method can be performed using robust regression (in particular, MM-estimation using Tukey's bisquare objective function) rather than standard linear regression:

```
library(robustbase)
betaIVW.robust             = summary(lmrob(betaYG~betaXG-1, weights=sebetaYG^-2, k.max=500))$coef[1]
sebetaIVW.robust.fixed     = summary(lmrob(betaYG~betaXG-1, weights=sebetaYG^-2, k.max=500))$coef[1,2]/
                             summary(lmrob(betaYG~betaXG-1, weights=sebetaYG^-2, k.max=500))$sigma
sebetaIVW.robust.random    = summary(lmrob(betaYG~betaXG-1, weights=sebetaYG^-2, k.max=500))$coef[1,2]/
                         min(summary(lmrob(betaYG~betaXG-1, weights=sebetaYG^-2, k.max=500))$sigma,1)
```

This is another alternative robust analysis method based on summarized data that downweights the contribution of heterogeneous genetic variants to the analysis model by capping the contribution to the objective function in the regression analysis from any single datapoint. See Burgess et al. [41] for more details.

Correlated variants:

If the genetic variants are correlated, these correlations should be accounted for in the analysis using generalized weighted linear regression:

```
Omega                       = sebetaYG%o%sebetaYG*rho
betaIVW.correl              = solve(t(betaXG)%*%solve(Omega)%*%betaXG)*t(betaXG)%*%solve(Omega)%*%betaYG
seIVW.correl.fixed          = sqrt(solve(t(betaXG)%*%solve(Omega)%*%betaXG))
residIVW                    = betaYG-betaIVW.correl*betaXG
sebetaIVW.correl.random     = sqrt(solve(t(betaXG)%*%solve(Omega)%*%betaXG))*
                          max(sqrt(t(residIVW)%*%solve(Omega)%*%residIVW/(length(betaXG)-1)),1)
betaEGGER.correl            = solve(t(cbind(rep(1, length(betaXG)), betaXG))%*%solve(Omega)%*%
                                    cbind(rep(1, length(betaXG)), betaXG))%*%
                                  t(cbind(rep(1, length(betaXG)), betaXG))%*%solve(Omega)%*%betaYG
            # first component is intercept term, second component is slope term (causal estimate)
residEGGER                  = betaYG-betaEGGER.correl[1]-betaEGGER.correl[2]*betaXG
varEGGER.correl.random      = solve(t(cbind(rep(1, length(betaXG)), betaXG))%*%solve(Omega)%*%
                                cbind(rep(1, length(betaXG)), betaXG))*
                        max(sqrt(t(residEGGER)%*%solve(Omega)%*%residEGGER/(length(betaXG)-2)),1)
seinterEGGER.correl.random = sqrt(varEGGER.correl.random[1,1])
sebetaEGGER.correl.random  = sqrt(varEGGER.correl.random[2,2])
```

The matrix `rho` comprises the pairwise correlations between the genetic associations (in particular, the genetic associations with the outcome). Provided that are genetic associations estimated in the same participants, these are equal to the correlations between the genetic variants themselves.

The generalized weighted linear regression can also be performed using the standard linear regression command after weighting the data by a Cholesky decomposition:

```
Omega    = sebetaYG%o%sebetaYG*rho
c_betaXG = solve(t(chol(Omega)))%*%betaXG
c_betaYG = solve(t(chol(Omega)))%*%betaYG
c_inter  = solve(t(chol(Omega)))%*%rep(1, length(betaXG))
betaIVW.correl              = lm(c_betaYG~c_betaXG-1)$coef[1]
sebetaIVW.correl.fixed      = sqrt(1/(t(betaXG)%*%solve(Omega)%*%betaXG))
sebetaIVW.correl.random     = sqrt(1/(t(betaXG)%*%solve(Omega)%*%betaXG))*
                        max(summary(lm(c_betaYG~c_betaXG-1))$sigma,1)
interEGGER.correl           = lm(c_betaYG~c_inter+c_betaXG-1)$coef[1]
betaEGGER.correl            = lm(c_betaYG~c_inter+c_betaXG-1)$coef[2]
seinterEGGER.correl.random = sqrt(solve(t(cbind(rep(1, length(betaXG)), betaXG))%*%solve(Omega)%*%
                                cbind(rep(1, length(betaXG)), betaXG))[1,1])*
                        max(summary(lm(c_betaYG~c_inter+c_betaXG-1))$sigma,1)
sebetaEGGER.correl.random  = sqrt(solve(t(cbind(rep(1, length(betaXG)), betaXG))%*%solve(Omega)%*%
                                cbind(rep(1, length(betaXG)), betaXG))[2,2])*
                        max(summary(lm(c_betaYG~c_inter+c_betaXG-1))$sigma,1)
```

The value of the intercept differs between the two versions of the method, but the same causal estimate is same in both cases.

Outlier detection (Cook's distance and Studentized residuals):

Cook's distance and Studentized residuals can be calculated for each genetic variant in the analysis:

```
cooks.distance(lm(betaYG~betaXG-1, weights=sebetaYG^-2))
rstudent(lm(betaYG~betaXG-1, weights=sebetaYG^-2)) # IVW method
cooks.distance(lm(betaYG~betaXG, weights=sebetaYG^-2))
rstudent(lm(betaYG~betaXG, weights=sebetaYG^-2))   # MR-Egger method
```

Plots of various diagnostic tools for detecting outliers and influential points (including Cook's distance against standardized residuals) can be produced by the command `plot(lm(betaYG~betaXG-1, weights=sebetaYG^-2))` or similar.

A.2 Consistency of IVW and MR-Egger methods and the InSIDE assumption

The genetic association with the outcome can be decomposed into the sum of a direct (pleiotropic) effect and an indirect (causal) effect:

$$\beta_{Yj} = \alpha_j + \theta \beta_{Xj} \tag{7}$$

where α_j is the effect of the genetic variant on the outcome that is not mediated via the risk factor of interest, and θ is the causal effect of the risk factor on the outcome.

For each genetic variant G_j, the ratio causal estimate is equal to:

$$\frac{\hat{\beta}_{Yj}}{\hat{\beta}_{Xj}} \xrightarrow{N\to\infty} \frac{\beta_{Yj}}{\beta_{Xj}} = \frac{\alpha_j + \theta\beta_{Xj}}{\beta_{Xj}} = \theta + \frac{\alpha_j}{\beta_{Xj}}, \tag{8}$$

where N is the sample size. A valid instrumental variable has no direct effect on the outcome ($\alpha_j = 0$), so the ratio estimate for a valid instrument is consistent for the causal effect θ. The IVW estimate is a weighted average of the ratio estimates, and so is also consistent for the causal effect when all instruments are valid.

More generally, the IVW estimate is:

$$\frac{\sum_j \hat{\beta}_{Yj}\hat{\beta}_{Xj}\,\mathrm{se}(\hat{\beta}_{Yj})^{-2}}{\sum_j \hat{\beta}_{Xj}^2\,\mathrm{se}(\hat{\beta}_{Yj})^{-2}} \xrightarrow{N\to\infty} \theta + \frac{\sum_j \alpha_j\beta_{Xj}\,\mathrm{se}(\hat{\beta}_{Yj})^{-2}}{\sum_j \beta_{Xj}^2\,\mathrm{se}(\hat{\beta}_{Yj})^{-2}}. \tag{9}$$

Therefore the asymptotic bias of the IVW estimate is zero if $\sum_j \alpha_j\beta_{Xj}\,\mathrm{se}(\hat{\beta}_{Yj})^{-2} = 0$; otherwise the IVW estimate is a biased estimate of the causal effect.

The MR-Egger estimate is a consistent estimate of the causal effect under the condition that the pleiotropic effects of the genetic variants α_j are uncorrelated with the associations of the genetic variants with the exposure β_{Xj} [9]. Specifically, we require the weighted covariance $\mathrm{cov}_w(\boldsymbol{\alpha}, \boldsymbol{\beta}_X)$ to be zero:

$$\mathrm{cov}_w(\boldsymbol{\alpha}, \boldsymbol{\beta}_X) = \frac{\sum_j (\alpha_j - \bar{\alpha}_w)(\beta_{Xj} - \bar{\beta}_{Xw})\,\mathrm{se}(\hat{\beta}_{Yj})^{-2}}{\sum_j \mathrm{se}(\hat{\beta}_{Yj})^{-2}} = 0 \tag{10}$$

where $\bar{\beta}_{Xw}$ is the weighted mean of the β_{Xj}, $\bar{\alpha}_w$ is the weighted mean of the α_j, and bold symbols represent vectors across the genetic variants. The slope coefficient from the weighted regression analysis is:

$$\hat{\theta}_{1E} = \frac{\mathrm{cov}_w(\hat{\boldsymbol{\beta}}_Y, \hat{\boldsymbol{\beta}}_X)}{\mathrm{var}_w(\hat{\boldsymbol{\beta}}_X)} \xrightarrow{N\to\infty} \frac{\mathrm{cov}_w(\boldsymbol{\beta}_Y, \boldsymbol{\beta}_X)}{\mathrm{var}_w(\boldsymbol{\beta}_X)} = \frac{\mathrm{cov}_w(\boldsymbol{\alpha}, \boldsymbol{\beta}_X)}{\mathrm{var}_w(\boldsymbol{\beta}_X)} + \theta\frac{\mathrm{cov}_w(\boldsymbol{\beta}_X, \boldsymbol{\beta}_X)}{\mathrm{var}_w(\boldsymbol{\beta}_X)} \tag{11}$$

which is equal to θ under the InSIDE assumption, where var_w is the weighted variance function.

Under the InSIDE assumption, the intercept term in the MR-Egger analysis can be interpreted as the average pleiotropic effect of the genetic variants included in the

analysis. The intercept term is zero when there is balanced pleiotropy (that is, the weighted average pleiotropic effect $\bar{\alpha}_w = \dfrac{\sum_j \alpha_j \operatorname{se}(\hat{\beta}_{Yj})^{-2}}{\sum_j \operatorname{se}(\hat{\beta}_{Yj})^{-2}}$ is zero) and the InSIDE assumption is satisfied. In this case, the MR-Egger and IVW estimates will coincide, and the IVW estimate will also be consistent; these two conditions imply that the bias term for the IVW estimate $\sum_j \alpha_j \beta_{Xj} \operatorname{se}(\hat{\beta}_{Yj})^{-2}$ is zero.

As a technical aside, in the original description of MR-Egger, the InSIDE assumption was presented as the independence between the distribution of pleiotropic effects and the distribution of associations of the genetic variants with the exposure [9]. This is a population version of the InSIDE assumption, and requires the genetic variants to be conceptualized as being sampled from a population of genetic variants. Consistency of the MR-Egger estimate under the population version of the InSIDE assumption requires the number of instruments to tend to infinity. In contrast, the version of the InSIDE assumption used in the proofs of consistency above is that the weighted covariance between the pleiotropic effects and associations of the genetic variants with the exposure is zero for the particular set of genetic variants in the analysis; this is a finite-sample version of the InSIDE assumption. This distinction also affects the definition of balanced and directional pleiotropy: in the population version, balanced pleiotropy is defined as the weighted mean of the distribution of pleiotropic effects equalling zero; in the finite-sample version, balanced pleiotropy is defined as the weighted mean of the pleiotropic effects equalling zero for the genetic variants included in the analysis.

A.3 Additional detail on applied examples

Plasma urate and coronary heart disease risk: Web Table A1 provides the genetic variants taken from White et al. [10] and their associations with plasma urate (in standard deviation units) and coronary heart disease risk (log odds ratios). These associations are displayed graphically in Figure 2 (right panel). Associations with plasma urate are taken from White et al.; associations with coronary heart disease risk are taken from the CARDIoGRAMplusC4D consortium 2015 data release [23] (data available at www.caridogramplus4d.org). This differs slightly from the genetic associations with coronary heart disease risk used by White et al., who used data from the CARDIoGRAMplusC4D consortium 2013 data release meta-analysed with data from the UCLEB consortium. This table is adapted from White et al. (Supplementary Table S3).

Genetic variant	Chr	Position	Gene region	Effect allele	Plasma urate Beta (SD units)	SE	Coronary heart disease Beta (log odds ratio)	SE
rs1471633	1	144435096	PDZK1	A	0.0568	0.0050	0.01709	0.00960
rs1260326	2	27584444	GCKR	T	0.0693	0.0049	-0.00326	0.00962
rs12498742	4	9553150	SLC2A9	A	0.3600	0.0051	0.01193	0.01112
rs2231142	4	89271347	ABCG2	T	0.1896	0.0077	0.02353	0.01490
rs675209	6	7047083	RREB1	T	0.0556	0.0059	0.01584	0.01027
rs1165151	6	25929595	SLC17A1	T	0.0779	0.0042	0.01636	0.00940
rs1171614	10	61139544	SLC16A9	T	0.0790	0.0070	0.01200	0.01242
rs2078267	11	64090690	SLC22A11	T	0.0732	0.0058	-0.00110	0.00980
rs478607	11	64234639	NRXN2	A	0.0264	0.0056	-0.00546	0.01276
rs3741414	12	56130316	INHBC	T	0.0649	0.0068	0.01213	0.01216
rs11264341	1	153418117	TRIM46	T	0.050	0.006	0.01689	0.00970
rs17050272	2	121022910	INHBB	A	0.035	0.006	-0.00599	0.01026
rs6770152	3	53075254	SFMBT1	T	0.044	0.005	0.01904	0.00932
rs17632159	5	72467238	TMEM171	C	0.039	0.006	0.00274	0.01027
rs729761	6	43912549	VEGFA	T	0.047	0.006	0.01258	0.01120
rs1178977	7	72494985	BAZ1B	A	0.047	0.007	0.00646	0.01256
rs10480300	7	151036938	PRKAG2	T	0.035	0.006	0.01703	0.01144
rs2941484	8	76641323	HNF4G	T	0.044	0.005	-0.00992	0.00930
rs10821905	10	52316099	A1CF	A	0.057	0.007	0.02326	0.01214
rs642803	11	65317196	OVOL1	T	0.036	0.005	0.03611	0.00935
rs653178	12	110492139	ATXN2	T	0.035	0.005	0.06443	0.01037
rs1394125	15	73946038	UBE2Q2	A	0.043	0.006	-0.00616	0.01073
rs6598541	15	97088658	IGF1R	A	0.043	0.006	0.00611	0.00945
rs7193778	16	68121391	NFAT5	T	0.046	0.008	0.00933	0.01360
rs7188445	16	78292488	MAF	A	0.032	0.005	-0.00686	0.01056
rs7224610	17	50719787	HLF	A	0.042	0.005	-0.00601	0.00975
rs742132	6	25715550	LRRC16A	A	0.054	0.0092	0.00863	0.01006
rs2307394	2	148432898	ORC4L	T	0.029	0.005	0.03780	0.00994
rs17786744	8	23832951	STC1	A	0.029	0.005	0.00521	0.00998
rs2079742	17	56820479	BCAS3	T	0.043	0.008	0.02360	0.01214
rs164009	17	71795264	QRICH2	A	0.028	0.005	0.01841	0.00943

Web Table A1: Genetic variants in different gene regions: genetic variant from White et al. [10], chromosome (Chr), position and nearest gene region, effect allele, per allele associations with plasma urate (standard deviation [SD] units) and coronary heart disease risk (log odds ratio) with corresponding standard errors (SE).

High-density lipoprotein cholesterol and coronary heart disease risk: Figure 7 (reproduced here as Web Figure A1) shows associations with high-density lipoprotein (HDL) cholesterol taken from the Global Lipids Genetics Consortium (GLGC) [50] and with coronary heart disease (CHD) risk taken from the CARDIoGRAM consortium [51] from all genetic variants that were associated with HDL-cholesterol at a genome-wide level of significance ($p < 5 \times 10^{-8}$) in the GLGC dataset. Associations with both HDL-cholesterol and CHD risk were obtained from Do et al. [52]. The inverse-variance weighted (IVW) estimate (solid line) and MR-Egger estimate (dashed line) are also displayed. The IVW estimate suggests a protective effect of HDL-cholesterol on CHD risk, whereas the MR-Egger estimate is compatible with the null and directional pleiotropy is detected.

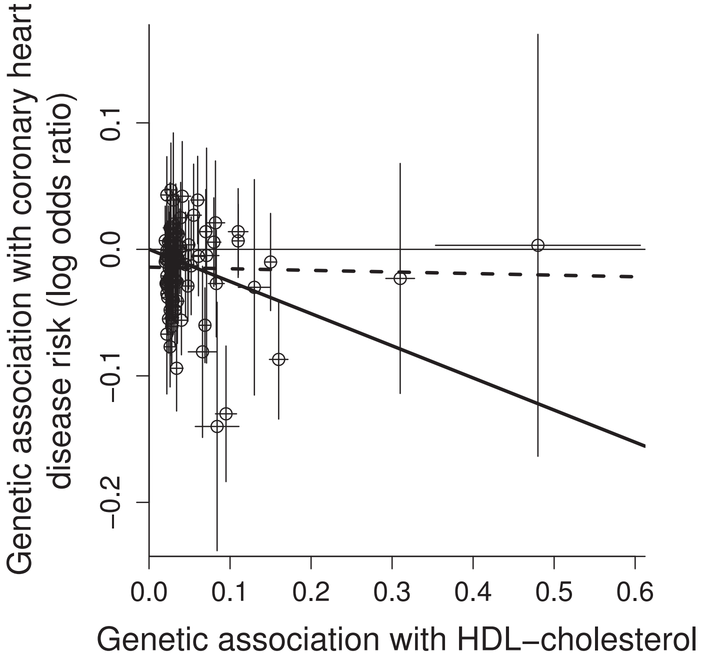

Web Figure A1: Graph showing further real example in which inverse-variance weighted estimate (solid line) and MR-Egger estimate (dashed line) differ substantially. Each point represents the per allele associations of a single genetic variant (lines from each point are 95% confidence intervals for the associations). Associations with HDL-cholesterol are in standard deviation units and associations with CHD risk are log odds ratios.

A.4 Finite-sample InSIDE assumption for uncorrelated and correlated variants

With a fixed number of uncorrelated genetic variants (hence finite-sample), consistency in the MR-Egger causal estimate requires the weighted covariance $\text{cov}_w(\boldsymbol{\alpha}, \boldsymbol{\beta}_X)$ to be zero, where:

$$\text{cov}_w(\boldsymbol{\alpha}, \boldsymbol{\beta}_X) = \frac{\sum_j (\alpha_j - \bar{\alpha}_w)(\beta_{Xj} - \bar{\beta}_{Xw})\,\text{se}(\hat{\beta}_{Yj})^{-2}}{\sum_j \text{se}(\hat{\beta}_{Yj})^{-2}} = 0$$

where $\bar{\beta}_{Xw}$ is the weighted mean of the β_{Xj}, $\bar{\alpha}_w$ is the weighted mean of the α_j, and bold symbols represent vectors across the genetic variants [41]. The slope coefficient from the weighted regression analysis is:

$$\hat{\theta}_{1E} = \frac{\text{cov}_w(\hat{\boldsymbol{\beta}}_Y, \hat{\boldsymbol{\beta}}_X)}{\text{var}_w(\hat{\boldsymbol{\beta}}_X)} \xrightarrow{N\to\infty} \frac{\text{cov}_w(\boldsymbol{\beta}_Y, \boldsymbol{\beta}_X)}{\text{var}_w(\boldsymbol{\beta}_X)} = \frac{\text{cov}_w(\boldsymbol{\alpha}, \boldsymbol{\beta}_X)}{\text{var}_w(\boldsymbol{\beta}_X)} + \theta \frac{\text{cov}_w(\boldsymbol{\beta}_X, \boldsymbol{\beta}_X)}{\text{var}_w(\boldsymbol{\beta}_X)}$$

With a fixed number of correlated genetic variants, coefficients from the MR-Egger generalized weighted linear regression are:

$$\begin{pmatrix} \hat{\theta}_{0E} \\ \hat{\theta}_{1E} \end{pmatrix} = [(\mathbf{1}\ \hat{\boldsymbol{\beta}}_X)^T \Omega^{-1} (\mathbf{1}\ \hat{\boldsymbol{\beta}}_X)]^{-1} (\mathbf{1}\ \hat{\boldsymbol{\beta}}_X)^T \Omega^{-1} \hat{\boldsymbol{\beta}}_Y$$

where $\mathbf{1}$ is a vector of 1s, and $(\mathbf{1}\,\hat{\boldsymbol{\beta}}_X)$ is a J by 2 matrix. The MR-Egger causal estimate tends (as the sample size increases to infinity) to:

$$\hat{\theta}_{1E} \xrightarrow{N\to\infty} \frac{(\boldsymbol{\beta}_X^T \Omega^{-1} \boldsymbol{\beta}_Y)(\mathbf{1}^T \Omega^{-1} \mathbf{1}) - (\boldsymbol{\beta}_X^T \Omega^{-1} \mathbf{1})(\boldsymbol{\beta}_Y^T \Omega^{-1} \mathbf{1})}{(\boldsymbol{\beta}_X^T \Omega^{-1} \boldsymbol{\beta}_X)(\mathbf{1}^T \Omega^{-1} \mathbf{1}) - (\boldsymbol{\beta}_X^T \Omega^{-1} \mathbf{1})(\boldsymbol{\beta}_X^T \Omega^{-1} \mathbf{1})}$$

Decomposing the genetic associations with the outcome into the causal and pleiotropic components ($\boldsymbol{\beta}_Y = \boldsymbol{\alpha} + \theta \boldsymbol{\beta}_X$), we obtain:

$$\hat{\theta}_{1E} \xrightarrow{N\to\infty} \theta + \frac{(\boldsymbol{\beta}_X^T \Omega^{-1} \boldsymbol{\alpha})(\mathbf{1}^T \Omega^{-1} \mathbf{1}) - (\boldsymbol{\beta}_X^T \Omega^{-1} \mathbf{1})(\boldsymbol{\alpha}^T \Omega^{-1} \mathbf{1})}{(\boldsymbol{\beta}_X^T \Omega^{-1} \boldsymbol{\beta}_X)(\mathbf{1}^T \Omega^{-1} \mathbf{1}) - (\boldsymbol{\beta}_X^T \Omega^{-1} \mathbf{1})(\boldsymbol{\beta}_X^T \Omega^{-1} \mathbf{1})}$$

This is equal to θ if:

$$(\boldsymbol{\beta}_X^T \Omega^{-1} \boldsymbol{\alpha})(\mathbf{1}^T \Omega^{-1} \mathbf{1}) - (\boldsymbol{\beta}_X^T \Omega^{-1} \mathbf{1})(\boldsymbol{\alpha}^T \Omega^{-1} \mathbf{1}) = 0.$$

This condition can also be written as:

$$\sum_{j_1} \sum_{j_2} \beta_{Xj1} \alpha_{j2} \Psi_{j1,j2} \times \sum_{j_1} \sum_{j_2} \Psi_{j1,j2} - \sum_{j_1} \sum_{j_2} \beta_{Xj1} \Psi_{j1,j2} \times \sum_{j_1} \sum_{j_2} \alpha_{j1} \Psi_{j1,j2} = 0,$$

where $\Psi = \Omega^{-1}$. When the variants are uncorrelated, only the diagonal elements of Ω and Ψ are non-zero, and these conditions simplify to the same weighted covariance as above.
